# Fully Biobased Biodegradable Elastomeric Polymer Blends Based on PHAs

**DOI:** 10.3390/polym17212811

**Published:** 2025-10-22

**Authors:** Pavol Alexy, Vojtech Horváth, Roderik Plavec, Zuzana Vanovčanová, Katarína Tomanová, Michal Ďurfina, Mária Fogašová, Leona Omaníková, Slávka Hlaváčiková, Zuzana Kramárová, Jana Navrátilová, Vojtěch Komínek, David Jaška, Jozef Feranc

**Affiliations:** 1 Institute of Natural and Synthetic Polymers, Faculty of Chemical and Food Technology, Slovak University of Technology in Bratislava, Radlinského 9, 812 37 Bratislava, Slovakia; 2 Department of Polymer Engineering, Faculty of Technology, Tomas Bata Univerzity in Zlin, Vavreckova 5669, 760 01 Zlin, Czech Republic; j1navratilova@utb.cz (J.N.); v_kominek@utb.cz (V.K.);

**Keywords:** biodegradable polymer blends, polyhydroxyalkanoates, elastomeric properties

## Abstract

This study examines binary blends of three types of polyhydroxyalkanoates (PHAs)—poly(3-hydroxybutyrate) (PHB), poly(3-hydroxybutyrate-co-3-hydroxyhexanoate) (PHBH), and poly(3-hydroxybutyrate-co-4-hydroxybutyrate) (P3HB4HB)—with a focus on their rheological, thermal, and mechanical behavior. The blends exhibit partial miscibility in both the melt and solid states. Glass transition analysis revealed that semicrystalline/amorphous PHA combinations are fully miscible (single Tg) at amorphous PHA contents below 30 wt%. Above this threshold, a two-phase morphology develops, consisting of crystalline spherulites embedded in an amorphous matrix. When the amorphous PHA content reached ≥30 wt%, the blends could be oriented by stretching, yielding materials that display thermoplastic elastomer (TPE)-like behavior without chemical modification of the base polymers. Thermal and mechanical characterization, supported by X-ray diffraction of samples before and after orientation, confirmed that the elastomeric properties originate from the multiphase architecture formed by crystalline and amorphous domains interconnected through a miscible amorphous fraction.

## 1. Introduction

Biodegradable, bio-based polymers are among the most promising solutions to mitigate the environmental impact of plastic waste. Conventional plastics contribute not only to large-scale pollution but also to the formation of microplastics, which result from the fragmentation of larger objects under mechanical, chemical, and environmental stresses [1]. Rubber-based products, including tires and common rubber goods, are also recognized as major sources of polymeric microparticles [2,3].

Efforts to reduce plastic pollution have largely focused on recycling; however, equal attention must be directed toward developing biodegradable materials from renewable resources. The packaging sector, responsible for nearly half of all plastic waste [4], has been the primary target of such research. Biopolymers such as polylactic acid (PLA), polyhydroxyalkanoates (PHAs), and polybutylene succinate (PBS) [5] are widely used, either alone or in blends, often with additives to improve performance [6]. Despite their potential, PLA and many PHAs—particularly polyhydroxybutyrate (PHB)—suffer from brittleness and low flexibility. These deficiencies are commonly addressed by incorporating plasticizers or blending with ductile petroleum-based polymers such as polybutylene adipate terephthalate (PBAT) [7,8,9] or polycaprolactone (PCL) [10,11]. While such strategies improve processability and toughness, they reduce the ecological value of the resulting products and, in many cases, limit biodegradability to industrial composting conditions.

Within the family of renewable biopolymers, PHAs are particularly promising because they are biotechnologically produced, fully biodegradable in diverse environments, and degrade into non-toxic products. PHB and its copolymer PHBV are the most extensively studied members, while more recent efforts have expanded to other PHA copolymers such as poly(3-hydroxybutyrate-co-4-hydroxybutyrate) (P3HB4HB) and poly(3-hydroxybutyrate-co-3-hydroxyhexanoate) (P3HB3HH) [12,13,14,15]. These materials, in addition to their established biomedical applications, show growing potential in packaging and related sectors. In recent years, many papers are focused on polymer blends, including blends of biodegradable and/or bio-based polymers, including PHAs [9,15]. Blending of various types of these polymers creates new materials with balanced properties. In the case of PHB this mainly takes the form of suppressing brittleness, while in some cases it manifests as a reduction in price (blends with TPS for example).

Beyond thermoplastics, research has also explored elastomeric materials derived from renewable polymers, often through copolymerization, grafting, crosslinking, or dynamic vulcanization. Examples include PBS copolymers with fatty diacids [5], crosslinked renewable polyesters [16], bio-based polyurethanes [17], reactive PLA/PHA blends [18], triblock copolymers incorporating PLA [19], and grafted systems such as β-myrcene/PLA [20] or natural rubber/PHA [21]. Dynamic vulcanization has been particularly studied for producing thermoplastic vulcanizates (TPVs), such as PLA/ENR [22,23], PLA/NR [24], and PLA/unsaturated polyester blends [25]. Although these systems improve toughness or elasticity, they often require chemical modification or complex processing, and their structure–property relationships remain incompletely understood [26,27,28,29,30].

Bio-based and biodegradable thermoplastic elastomers (TPEs) are now attracting increasing attention as sustainable alternatives to petroleum-derived elastomers. Some materials are both bio-based and biodegradable, while others meet only one of these criteria. Applications already include medical devices [31,32], sports footwear [33,34], apparel fibers [35], and 3D printing filaments [36].

Most reported bio-based elastomer systems, however, still depend on chemical modification, copolymerization, or complex processing, which limits both their scalability and sustainability. To overcome these drawbacks, a more straightforward strategy is needed—one that maintains full biodegradability under natural conditions while providing elastomeric properties.

The objective of this study was therefore to design and evaluate binary blends of commercially available PHAs capable of exhibiting TPE-like behavior without requiring chemical modification, copolymerization, or dynamic vulcanization. Building on the work of Ďurfina et al. [37], who demonstrated flexible PHA blends for 3D printing, we extend this approach by systematically investigating combinations of semicrystalline and amorphous PHAs. The elastomeric properties of such blends arise from their unique phase morphology: crystalline domains provide strength, while amorphous domains impart flexibility. Partial miscibility in the amorphous regions and the formation of a two-phase system are essential to enable reversible elastic deformation while suppressing irreversible plastic flow [38].

In this work, binary PHA blends spanning a wide range of mechanical properties were prepared by melt blending and comprehensively characterized. Their rheological, thermal, mechanical, and morphological properties were analyzed to assess their potential as sustainable and fully biodegradable thermoplastic elastomers. Unlike previous studies that relied on chemical modification, copolymerization, or complex reactive processing, this work employs a simple physical blending approach using commercially available PHAs. The novelty of this study lies in demonstrating that partial miscibility between semicrystalline and amorphous PHA phases can be exploited to achieve thermoplastic elastomer-like behavior without altering the chemical structure of the polymers. This highlights a new design pathway for developing fully biodegradable elastomeric materials based solely on physical interactions and phase morphology control.

## 2. Materials and Methods

### 2.1. Materials

Three types of Polyhydroxyalkanoates (PHAs) were delivered by the company Panara a.s., Nitra, Slovakia: PHA1—polyhydroxyalkanoate P3HB with high crystallinity (52%), Tg = 5.1 °C, Tm = 166.7 °C, Ð = 3.1 and Mw 219,000 g/mol, in powder form. PHA2—amorphous polyhydroxyalkanoate P3HB4HB with 0% crystallinity, Tg = −14.9 °C, Ð = 4.0 and Mw 250,000 g/mol, Tm not detectable on DSC. PHA3—polyhydroxyalkanoate P3HB3HH with medium crystallinity (36.2%), Tg = 2.4 °C, Tm = 148.6 °C, Ð = 3.8 and Mw 230,000 g/mol.

### 2.2. Methods

#### 2.2.1. Blend Preparation

All blending components were introduced into the hopper of a laboratory twin-screw extruder (Labtech, Thailand) with a screw diameter of 16 mm and a length-to-diameter (L/D) ratio of 40. The screw geometry incorporated three kneading zones, and atmospheric venting was positioned at the 38D mark on the barrel. Extrusion was conducted at a screw speed of 150 RPM, with the temperature profile along the barrel set to 70–120–170–180–190–190–190–180–175–170 °C from the hopper to the die.

The extruded melt was cooled in a water bath maintained at 20 °C. After exiting the water bath, surface moisture was removed by vacuum suction, and the strand was pelletized using a pelletization cutter. The pellets were then dried in a hot air oven at 50 °C for 24 h before further processing.

#### 2.2.2. Procedure for the Preparation of Monofilaments

Prepared pellets of individual blends were introduced into the hopper of laboratory Brabender single-screw extruder with screw parameters D = 19 mm, L/D = 25 with a compression ratio of 1:3. During the preparation of monofilaments with a diameter of 1 mm, the following temperature profile was set in the direction hopper-head: 190–180–170–160 °C. Through a nozzle with a circular diameter of 1.8 mm, the polymer melt was extruded into a tempering bath with a water temperature of 30 °C. After exiting the filament from the water bath, the filament was freed from surface moisture by passing the filament through a slot connected to a vacuum.

#### 2.2.3. Procedure for Drawing Monofilaments to the Maximum Drawing Ratio

The extruded filaments were oriented by drawing to the maximum achievable draw ratio using a KMS-PT Ltd, Prešov, Slovakia. drawing device equipped with three feed rollers and three take-up rollers. The draw ratio was controlled by adjusting the relative speeds of the feed and take-up rollers. Filament stretching occurred over a 1 m path within a water bath maintained at 80 °C. The draw ratio (DR) was defined as:

(1) $$\lambda =\frac{l_{max}}{l_{0}}$$

where

$l_{max}$ is maximum length of drawn sample
$l_{0}$ initial length of sample before drawing
Value λ = 1 means that sample was not possible to stretch.

#### 2.2.4. Rheological Properties

Rheological properties were measured using an RPA 2000 (Alpha Technologies, Hudson, OH, USA) oscillating rheometer. The samples were placed in the biconical chamber of the oscillatory rheometer, and the testing program was initiated. After sealing the chamber, the temperature was set to 180 °C, and the samples were preheated for 1.5 min at an oscillation angle of 6° and an oscillation frequency of 60 CPM. Following the preheating stage, the temperature was adjusted to 160 °C, and the flow curve measurement began at the same oscillation frequency of 60 CPM. The oscillation angle was gradually increased from 2° to 50°, resulting in shear rates ranging from 1.34 to 40 s^−1^. The complex viscosity was recorded using the rheometer software (Enterprise Online Manager software, Version 6.30.4, Alpha Technologies, Hudson, OH, USA), and the relationships between complex viscosity and shear rate were evaluated for all samples.

#### 2.2.5. Thermal Properties (DSC)

The thermal transitions of the blends were analyzed using a differential scanning calorimeter (DSC 1, Mettler-Toledo Inc., Greifensee, Switzerland). Approximately 10–15 mg of sample pellets were sealed in standard aluminum pans, with an empty aluminum pan used as the reference. Nitrogen was applied as a purge gas at a flow rate of 50 mL/min. The following parameters were determined: glass transition temperature (Tg), crystallization temperature (Tc), and melting temperature (Tm). The heating/cooling program was applied according to the measurement protocol described below.

- Isothermal hold at −70 °C for 3 min;
- First heating cycle: −70 °C to +200 °C at a heating rate of 10 °C/min;
- Isothermal hold at +200 °C for 3 min;
- Cooling cycle: +200 °C to −70 °C at a cooling rate of 10 °C/min;
- Isothermal hold at −70 °C for 3 min;
- Second heating cycle: −70 °C to +200 °C at a heating rate of 10 °C/min.

The heat flow dependency on temperature was recorded as the output of the measurement. All investigated thermal parameters were analyzed using DSC curves and processed with the SW STARe 16.40 evaluation software (Mettler-Toledo Inc.).

#### 2.2.6. Scanning Electron Microscopy (SEM)

The morphology of the samples was examined using scanning electron microscopy (SEM). To visualize crystalline structures, samples were etched by immersion in a 1% NaOH solution at 90 °C for 15 min. The etched samples were then rinsed with distilled water, dried in an oven at 60 °C for 24 h, and subsequently sputter-coated with a gold/palladium alloy using a Balzers SCD 050 coater. Micrographs were obtained with a JEOL F 7500 scanning electron microscope (JEOL, Tokyo, Japan).

#### 2.2.7. Mechanical Properties Measurement

##### Tensile Test

Tensile properties were measured on both as-extruded (undrawn) and drawn monofilaments. Tests were conducted in accordance with the general principles of ISO 527-1 [39], with specific adaptations from ASTM D3822 for single-filament testing. A Zwick-Roell universal testing machine (Zwick-Roell, Ulm, Germany) equipped with fiber grips was used, operating at a crosshead speed of 100 mm/min.

##### Hardness Testing

Hardness was evaluated on the same specimens used for bending tests. Measurements were performed according to ISO 868 [40], and values were recorded on the Shore D scale.

##### Determination of Elastically Reversible Deformation on Non-Stretched Specimen

**Figure 1:**
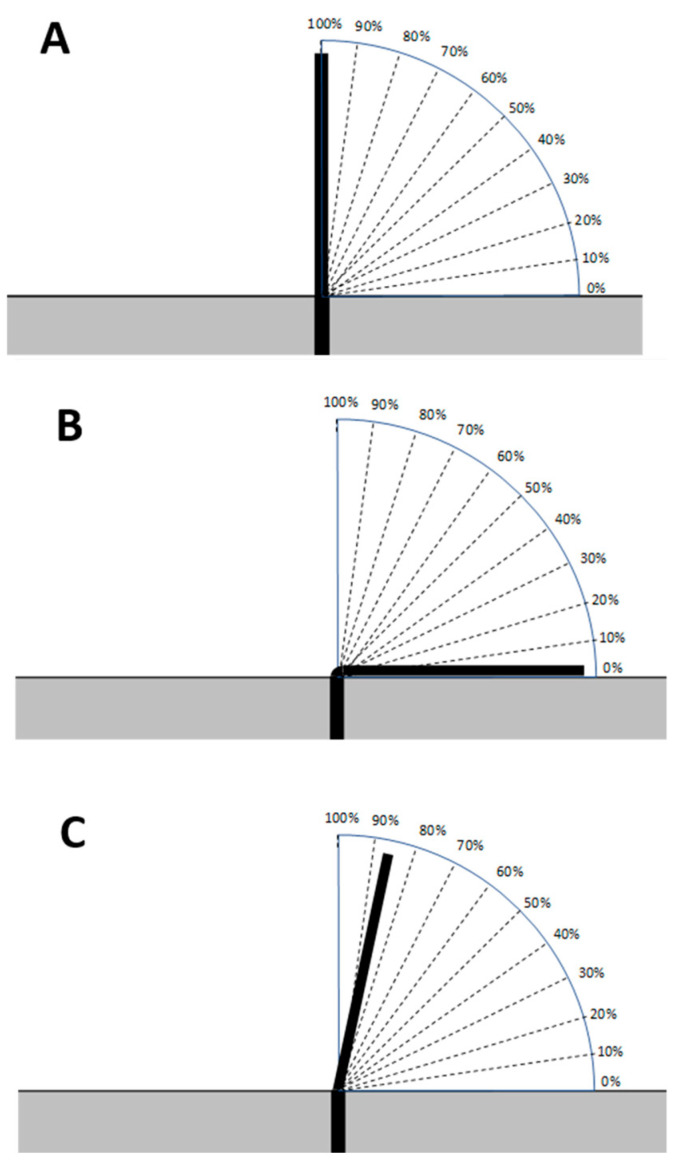
Schematic representation of the test used to determine elastically reversible deformation. (**A**)—initial position; (**B**)—specimen bent by 90°; (**C**)—specimen position after release.

Elastically reversible deformation on non-stretched specimens was determined using a proprietary method as elastic recovery at given conditions. Specimens were prepared in the 1BA geometry according to ISO 527 and fixed vertically by the wider end, forming a 90° angle with a horizontal support. Each specimen was bent by 90° into the horizontal position, maintained for 15 s, and then released. After a recovery period of 15 s, the degree of reversible deformation was recorded as the percentage return toward the initial vertical position. A schematic representation of the test setup is shown in Figure 1.

##### Determination of the Range of Elastically Reversible Deformation (RED) in Tension and E1 and E2 Moduli

**Figure 2:**
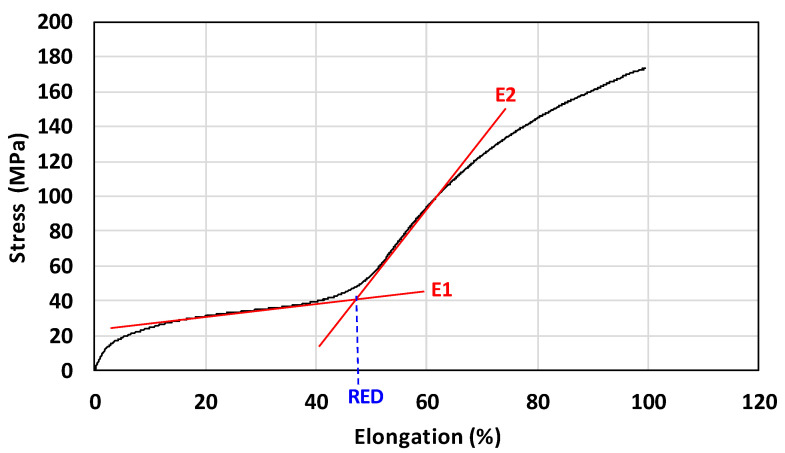
Representative tensile curve illustrating the method for determining the parameters RED, E1, and E2.

The range of elastically reversible deformation (RED) in tension, along with the tensile moduli E1 and E2, was determined as illustrated in Figure 2. Monofilaments prepared according to Section 2.2.2 and subsequently drawn as described in Section 2.2.3 were tested on a Zwick-Roell universal testing machine at a crosshead speed of 100 mm/min.

The tensile curve obtained from these measurements was divided into two characteristic regions. The initial linear region represents the elastically reversible deformation, while the subsequent region corresponds to stress increase beyond the elastic limit until rupture. The modulus E1 was calculated as the slope of the linear portion of the stress–strain curve within the elastically reversible range. The modulus E2 was defined as the slope of the linear region beyond the limit of elastic deformation, up to failure. From a practical point of view, modulus E1 represents the material’s resistance to deformation within the elastic deformation region; it is a parameter expressing the stiffness of the elastic material. Modulus E2, on the other hand, characterizes the resistance to deformation after the material reaches its maximum of elastic deformation and it is related to the stress which is necessary for its failure.

The RED value was determined as the strain coordinate corresponding to the intersection point of the two linear fits (E1 and E2). The procedure for defining these parameters is schematically shown in Figure 2.

It should be noted that the terms elastic recovery and range of reversible elastic deformation represent distinct material parameters. Elastic recovery (for non-stretched samples) represents the part of the elastically reversible deformation that can be quantitatively determined under the given testing conditions, whereas the maximum range of elastically reversible deformation cannot be directly determined. The range of reversible elastic deformation (RED for stretched samples) as defined by the method applied to stretched samples, represents the theoretical range of elastic deformation under the given conditions, which can be determined by extrapolating the two linear regions of the tensile stress–strain curve (see RED in Figure 2).

##### Preparation of Test Specimens by Injection Molding

Bar-shaped specimens (80 × 10 × 4 mm) were prepared by injection molding for hardness testing, while type 1BA specimens (ISO 527) were molded for the determination of elasticity in non-oriented systems. All specimens were produced on a BOY 60E injection molding machine (Dr. Boy GmbH & Co. KG, Neustadt-Fernthal, Germany). The processing parameters applied during injection molding were as follows:

- Injection volume: 18 cm^3^;
- Injection speed: 10 cm^3^/s;
- Injection pressure: 1400 bar;
- Holding pressure: 1200 bar;
- Holding time: 10 s;
- Cooling time: 15 s;
- Mould temperature: 50 °C;
- Barrel temperature profile (from hopper to nozzle): 160–170–175–170–170 °C.

#### 2.2.8. Determination of Surface Energy of Polymers

Surface energy was determined by contact angle measurement. Samples for measurement were prepared by hot pressing the polymer granulate in a LabEcon 600 hydraulic press (Fontijne Presses, Delft, The Netherlands) at the temperature of 180 °C and the pressure of 20 MPa. The material was preheated for 1 min and subsequently pressed for 2 min, followed by cooling to room temperature under pressure.

The contact angle was measured using a SEE System (Advex Instruments, Brno, Czech Republic), and the values were calculated with the SeeSystem 6.3 software. The measurements were expressed as average based on 10 individual measurements. Determination of total surface energy as well as their dispersive and polar part was done according to the methodology described in work [41]. Distilled water and 1-bromonaphthalene were used for contact angle measurement with values of dispersive as well as polar components as follows [42]:

$$\mathrm{Water}\;\gamma^{d}=21.8\;mN/m\;\;\;\gamma^{p}=51\;mN/m\;1\text{-}\mathrm{bromonaphthalene}\;\gamma^{d}=43.7\;mN/m\;\;\gamma^{p}=0.9\;mN/m$$

#### 2.2.9. Wide-Angle X-Ray Scattering

X-ray diffraction (XRD) patterns were recorded using a Dynamic 500 diffractometer (Anton Paar, Graz, Austria). Extruded tape specimens, both before and after orientation, were analyzed as representative samples. Measurements were conducted in Bragg–Brentano geometry with a Primux 3000 sealed-tube X-ray source equipped with a Cu anode and a Ni/C divergent-beam multilayer monochromator (wavelength λ = 0.154 nm) operating in reflection mode.

The diffraction data were collected over a 2θ range of 5–35° with a step size of 0.05°. The degree of crystallinity ($X_{C}$) was determined using XRD analysis software (Anton Paar, version XRD analysis 1.2.0.3511) by deconvolution of the crystalline (*A_C_*) and amorphous (*A_A_*) contributions. The crystallinity was calculated according to Equation (2):

(2) $$X_{C}=\frac{A_{C}}{A_{C}+A_{A}}\cdot 100\%$$

## 3. Results and Discussion

The primary aim of this study was to investigate the preparation of thermoplastic elastomers (TPEs) from renewable, bio-based polymers that are fully biodegradable under natural environmental conditions, including soil, freshwater, seawater, and both industrial and home composting. The approach deliberately avoided the synthesis of specialized copolymers or the use of reactive processing methods. Biodegradability of the resulting blends was ensured by selecting base polymers exclusively from the polyhydroxyalkanoate (PHA) family, which are known to degrade in all natural environments.

This work focused on blends combining amorphous and semicrystalline PHAs, formulated according to the following design principles:

(a) The blends must exhibit a two-phase morphological structure;
(b) The polymers must demonstrate partial miscibility in the amorphous phase.

Binary blends of selected polymers were prepared in accordance with the procedure described in Section 2.2.1, and their compositions are summarized in Table 1.

### 3.1. Rheology

Rheological measurements were performed to assess the miscibility of the polymer blends in the melt state and to relate these findings to the subsequent determination of glass transition temperatures (Tg) in the solid state. For completely miscible polymer pairs, the composition dependence of viscosity is expected to follow the logarithmic mixing rule, as expressed by Equation (3) [9,43,44]:

(3) $$ln\eta_{B}=\emptyset_{a}\cdot ln\eta_{a}+\emptyset_{b}\cdot ln\eta_{b}$$

where

$ln\eta_{B}$—viscosity of the polymer blend.
$ln\eta_{a}$—viscosity of component *a*.
$ln\eta_{b}$—viscosity of component *b*.
$\emptyset_{a}$, $\emptyset_{b}$—volume fractions of components *a* and *b*.

**Figure 3:**
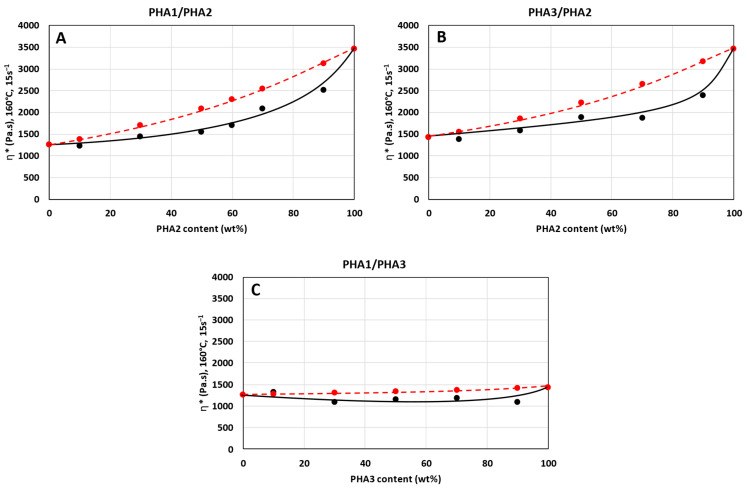
Complex viscosity of binary polymer blends at 160 °C and a shear rate of 15 s^−1^: (**A**) PHA1/PHA2, (**B**) PHA3/PHA2, and (**C**) PHA1/PHA3. Black points represent experimental values; red points represent calculated values according to the logarithmic mixing rule.

The graphical dependence of complex viscosity on the composition of the binary blends, measured according to the procedure described in Section 2.2.4, is presented in Figure 3 (black data points). For comparison, the values calculated using the additive logarithmic mixing rule (Equation (3)) are also shown (red data points).

The viscosity–composition profiles of the binary blends reveal pronounced deviations from the logarithmic additivity rule, particularly for the PHA1/PHA2 and PHA3/PHA2 systems. These pairs represent semicrystalline–amorphous combinations, which differ substantially in the viscosities of their individual components. By contrast, the PHA1/PHA3 system, involving two semicrystalline polymers with similar viscosities, exhibits smaller deviations.

**Figure 4:**
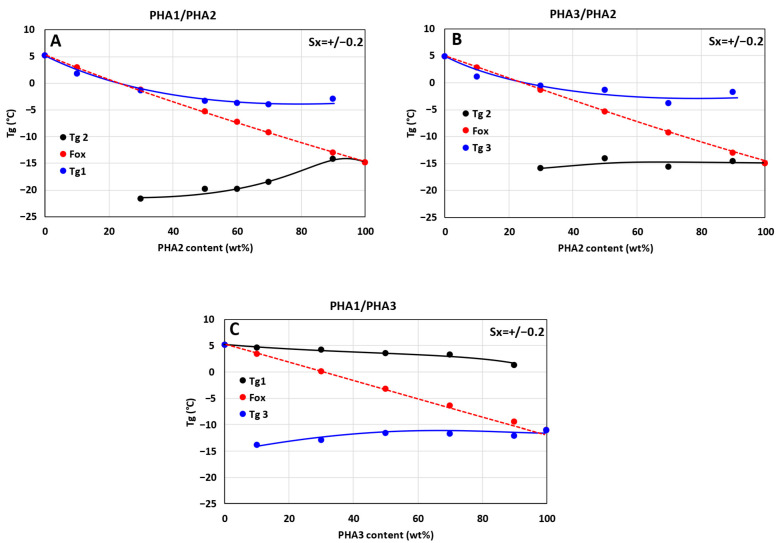
Dependence of Tg on the composition of binary polymer blends: (**A**) PHA1/PHA2, (**B**) PHA3/PHA2, and (**C**) PHA1/PHA3. The red symbols in the graphs represent Tg values calculated according to the Fox Equation (3).

Negative deviations from the logarithmic rule, i.e., lower values of the experimentally determined complex viscosity of binary blends compared to the values calculated according to Equation (3), can arise from several factors. Most commonly, they reflect intermolecular interactions between polymer segments, leading to plasticization effects. Another possibility is that differences in molecular weight may cause the lower-molecular-weight component to act as a lubricant; however, this explanation appears unlikely in the present case, as the polymers studied had comparable molecular weights. As shown in Figure 4 (Section 3.2), shifts in the Tg values of the blends indicate partial miscibility and mutual plasticization, which account for the observed rheological behavior.

These findings are consistent with earlier reports. Charon et al. [45] observed deviations from additivity in PHB blends with natural terpenes, while Bugnicourt et al. [46] emphasized in their review that PHA melt rheology is highly sensitive to blend composition and modifiers. Both studies highlight the role of intermolecular interactions, thermal sensitivity, and processing stability in determining rheological properties. In line with these observations, the negative deviations in viscosity reported here primarily reflect segmental interactions and plasticization, underscoring the importance of miscibility effects in the melt behavior of PHA-based systems.

### 3.2. Differential Scanning Calorimetry

To evaluate the miscibility of polymer pairs, differential scanning calorimetry (DSC) is frequently employed for the determination of glass transition temperatures (Tg). In immiscible blends, the DSC thermogram displays two distinct Tg values corresponding to the individual polymers, which remain independent of blend composition. In contrast, a completely miscible blend exhibits a single Tg that typically lies between the Tg values of the neat components and shifts with composition. Several theoretical models have been proposed to describe the dependence of Tg on blend composition, among which the Fox equation [47] is most widely applied (4):

(4) $$\frac{1}{T_{g}}=\frac{w_{1}}{T_{g1}}+\frac{w_{2}}{T_{g2}}$$

where

*w*_1_, *w*_2_—weight fractions of the polymers.
$T_{g1}$, $T_{g2}$—glass transition temperatures of the polymers in Kelvin.

For all prepared blends, thermal properties were measured by DSC. The Tg values were determined from the inflection point of the glass transition observed in the thermogram during the second heating cycle. The experimental Tg values of the blends, together with the theoretical predictions based on the Fox equation (Equation (4)), are presented in Figure 4.

From the Tg–composition dependencies it follows that the blends will have a rather complex morphological structure, particularly the blends of semicrystalline PHA with amorphous PHA (PHA1/PHA2 and PHA3/PHA2). Both combinations (Figure 4A,B) show miscibility of the semicrystalline PHAs (1 and 3) with the amorphous PHA2 up to 30% PHA2 in the blend. The dependencies for the blends PHA1/PHA2 and PHA3/PHA2 exhibit only a single Tg value up to 30% PHA2 content, which decreases according to the Fox equation. Above 30% PHA2 content, the decrease in the Tg of the crystalline PHAs levels off and does not change further, which indicates the formation of a phase consisting of PHA1 or PHA3 containing less than 30% PHA2; this blend is miscible without a phase separation. In other words, up to about 30%, PHA2 dissolves in PHA1 or PHA3. Therefore, below 30% PHA2 in the blend, the DSC thermograms did not show separate Tg transitions for PHA2, PHA1, or PHA3, but only a single common Tg value.

Above 30% PHA2 in the blend, phase separation occurs, forming a new phase composed of PHA2 with a small amount of dissolved PHA1 or PHA3. This is evident from the fact that from 30% PHA2 in the blends, a second Tg transition is detected on the DSC thermograms; however, this Tg does not correspond exactly to the Tg value of pure PHA2, but is slightly lower, particularly for the PHA1/PHA2 combination. For 30% PHA2 in the PHA1/PHA2 blend, the Tg of PHA2 decreases from −15 °C to −22 °C. With decreasing PHA1 content, the Tg of PHA2 gradually increases. The mutual solubility of the polymers PHA1/PHA2 and PHA3/PHA2 is also indicated by the viscosity changes shown in Figure 3, as discussed in Section 3.1.

For these two types of binary blends, the following morphology can therefore be assumed on the basis of the results discussed so far:

Up to 30% PHA2 in PHA1 or PHA3, the blend forms a single-phase system of completely miscible polymers.

Above 30% PHA2, phase separation occurs, with one phase formed by a miscible PHA1/PHA2 (or PHA3/PHA2) blend and the other phase formed by a miscible PHA2/PHA1 (or PHA2/PHA3) blend.

**Figure 5:**
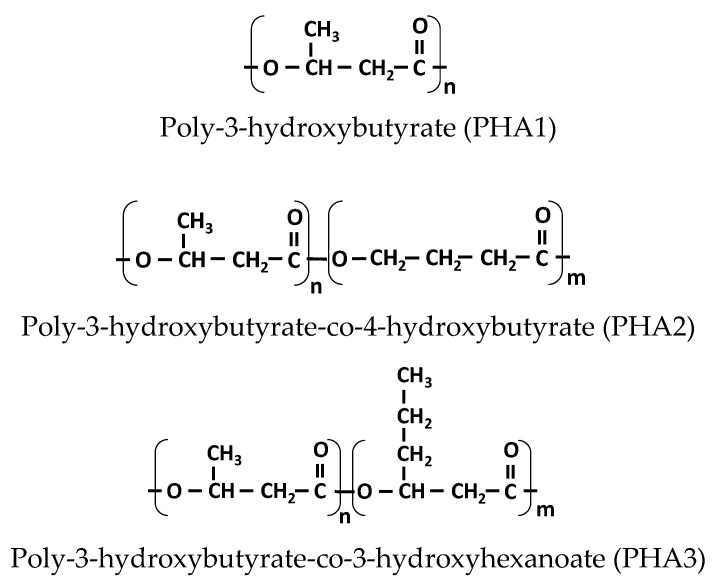
Structure of PHA1, PHA2, and PHA3.

From the dependencies shown in Figure 4A,B, it is clear that these effects are more pronounced for the PHA1/PHA2 combination than for the PHA3/PHA2 combination, which indicates that the miscibility of PHA1/PHA2 is better than that of PHA3/PHA2. It can be assumed that these differences are most likely caused by structural variations at the molecular level, since PHA3 contains longer side chains of the polyhydroxyhexanoate unit (CH_3_–CH_2_–CH_2_–), whereas PHA1 and PHA2 have only a CH_3_ group as a side substituent from the polyhydroxybutyrate unit, as illustrated in Figure 5.

The differences in thermal transitions, crystallinity, and miscibility observed among the investigated PHAs can be directly correlated with their molecular structures. PHA1 (poly-3-hydroxybutyrate) is a highly regular homopolymer containing short methyl side groups, which enable close chain packing and the formation of well-defined lamellae. Consequently, it exhibits high crystallinity and a relatively high melting temperature [48,49,50]. In contrast, PHA2 [poly(3-hydroxybutyrate-co-4-hydroxybutyrate)] incorporates flexible 4-hydroxybutyrate units that introduce irregularities along the polymer backbone, disrupting crystal packing and lowering both the degree of crystallinity and the glass transition temperature (Tg ≈ −15 °C) [51]. PHA3 [poly(3-hydroxybutyrate-co-3-hydroxyhexanoate)] contains longer side chains derived from the 3-hydroxyhexanoate units (–CH_2_–CH_2_–CH_3_), which further reduce packing efficiency and interchain interactions. Although PHA3 remains semicrystalline, its crystallinity is lower and its Tg is reduced relative to PHA1, reflecting increased segmental flexibility and weaker intermolecular forces [52,53].

These structural distinctions account for the experimental trends reported in Section 3.1, Section 3.2 and Section 3.3. The high regularity and crystallinity of PHA1 confer rigidity and strength, while the amorphous, flexible nature of PHA2 enhances ductility and elastic recovery. PHA3, exhibiting intermediate crystallinity, provides a balance between stiffness and flexibility. Accordingly, blends combining semicrystalline PHAs (PHA1 or PHA3) with amorphous PHA2 form biphasic systems in which partial miscibility within the amorphous domains facilitates stress transfer and elastic deformation. The molecular architecture of each PHA therefore governs its packing behavior, crystallization ability, and glass transition characteristics, which collectively determine the mechanical performance and thermoplastic elastomer-like properties of the resulting blends.

As for the combination of both semicrystalline PHAs (PHA1/PHA3), they are also partially miscible according to the Tg changes, but to a much lesser extent than their miscibility with PHA2. However, even in this case, a similar blend morphology as in the PHA1/PHA2 and PHA3/PHA2 blends can be assumed, with the miscibility region being significantly smaller and the mutual segmental interactions much weaker. For confirmation of our finding based on Tg evaluation, we also calculated interfacial tension $\gamma_{12}\;$ for all three types of binary PHAs blends, based on surface tension measurement of individual components [41]. The obtained values are listed in Table 2.

Very low values of interfacial tension for PHA3/PHA2 and PHA1/PHA2 combinations 0.015 mN/m and 0.077 mN/m, respectively, in contrast to much higher value for PHA1/PHA3 combination 2.103 mN/m indicate much better miscibility between PHA1/PHA2 and PHA3/PHA2 in comparison to miscibility of PHA1/PHA3. This calculation is in very good agreement with evaluation of miscibility based on Tg measurements.

A comparison between our findings and those reported in the recent literature reveals several unifying principles governing the structure–property relationships of PHA-based blends, while also highlighting differences arising from composition and experimental focus. Consistent with Majka et al. [54], we demonstrate that blends of semicrystalline PHB with amorphous PHA components exhibit partial miscibility, reflected in the presence of a single glass transition temperature (Tg) at lower amorphous contents. In both cases, crystallinity is governed solely by the semicrystalline phase, decreasing with increasing amorphous fraction. However, our work identifies a distinct miscibility threshold at ~30 wt% amorphous PHA, above which phase separation occurs, whereas the cited study suggests a more gradual transition. These results align with the broader principles outlined by Feijoo et al. [55], who emphasized that miscibility in the amorphous phase is a key determinant of thermal transitions and that the amorphous fraction does not co-crystallize but modulates crystallization kinetics and morphology. Our observations are further supported by the review of Bugnicourt et al. [46], which similarly stresses the link between amorphous miscibility and Tg behavior and highlights the role of amorphous domains as plasticizing agents rather than contributors to lamellar order.

**Figure 6:**
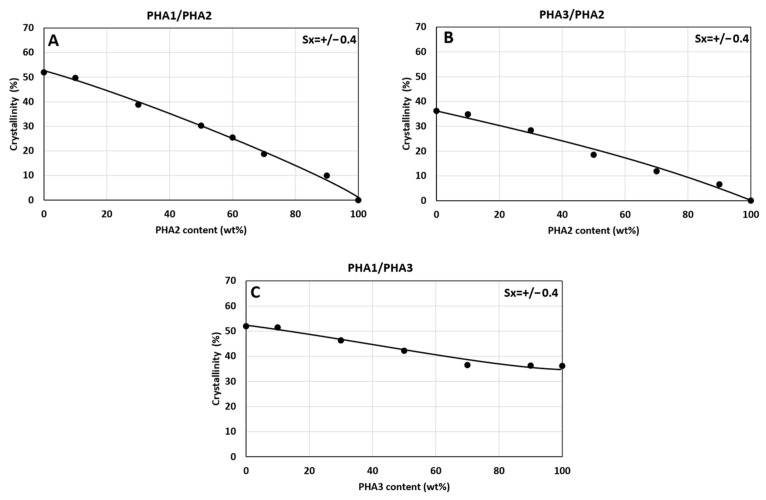
Dependence of crystallinity on the composition of binary polymer blends: (**A**) PHA1/PHA2, (**B**) PHA3/PHA2, and (**C**) PHA1/PHA3.

**Figure 7:**
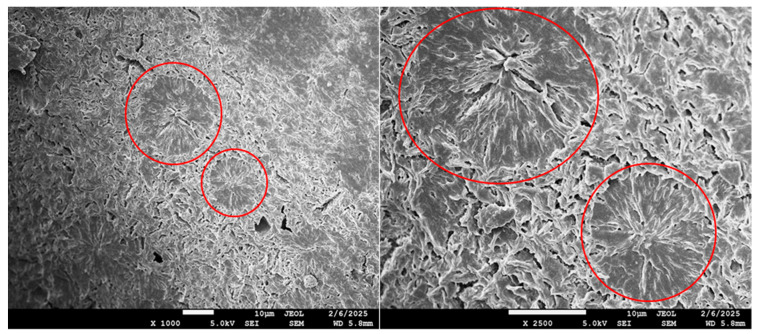
SEM photos of spherulites in morphology of blend B17 (PHA1/PHA2 = 50/50) at magnification 1000× and 2500×. For better illustration, some spherulites are marked with a red circle.

**Figure 8:**
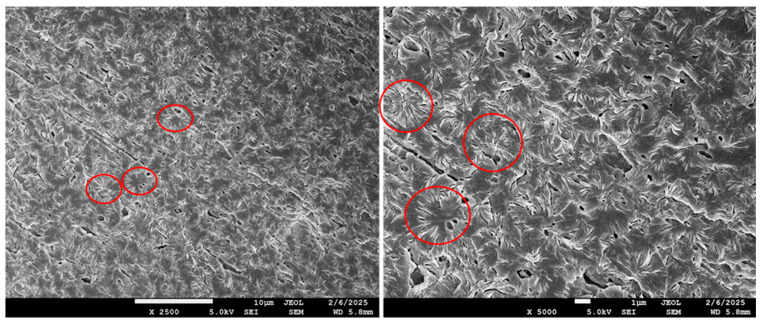
SEM photos of spherulites in morphology of blend B4 (PHA1/PHA3 = 50/50) at magnification 2500× and 5000×. For better illustration, some spherulites are marked with a red circle.

The crystallinity content, calculated based on the enthalpy from the second DSC heating run and the enthalpy value ΔHm^0^ = 146 J/g, varies practically linearly between the crystallinities of the components of the binary blends. The dependence of the crystalline phase content on the composition of the binary blends is shown in Figure 6. From this it follows that in the blends only the semicrystalline type of PHA crystallizes, and therefore the crystallinity content increases proportionally with the increasing content of semicrystalline PHAs. In the case of PHA1 or PHA3 in combination with amorphous PHA2 above 30% concentration, crystallites are formed in the blend structure in the form of isolated spherulites with a diameter of about 15–25 µm, as can be seen from the SEM micrograph (Figure 7). For the PHA1/PHA3 combination, i.e., for both semicrystalline polymers, the total crystallinity never falls below 35%, and the resulting crystallites in the form of spherulites create a dense, continuous crystalline phase with spherulite sizes of about 2–4 µm (Figure 8). Similar morphological structures have also been observed in other studies focusing on blends of various types of PHAs [56,57,58] or blends of PHA with other biodegradable polymers, e.g., PLA [59].

### 3.3. Mechanical Properties

#### 3.3.1. Mechanical Properties of Non-Stretched Samples

**Figure 9:**
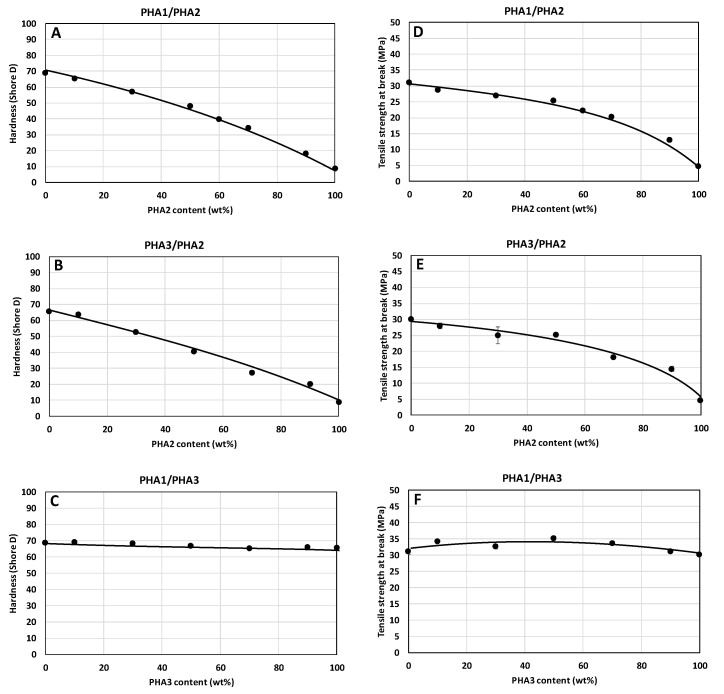
Dependence of hardness (**A**–**C**) and tensile strength (**D**–**F**) of non-oriented specimens on the composition of binary polymer blends: (**A**,**D**) PHA1/PHA2, (**B**,**E**) PHA3/PHA2, and (**C**,**F**) PHA1/PHA3.

**Figure 10:**
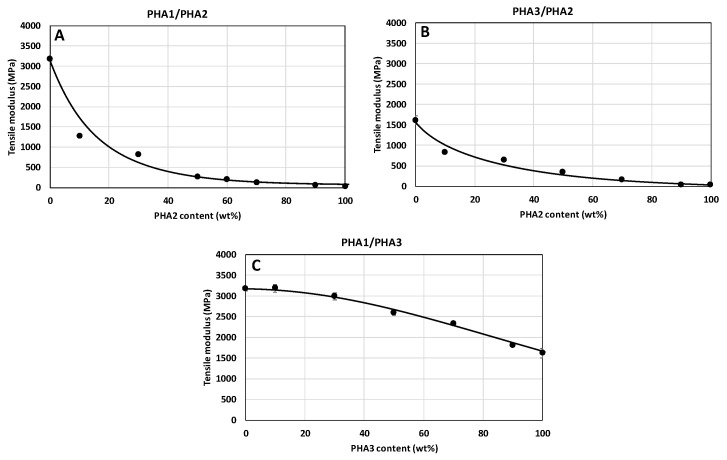
Dependence of the tensile modulus of non-oriented specimens on the composition of binary polymer blends: (**A**) PHA1/PHA2, (**B**) PHA3/PHA2, and (**C**) PHA1/PHA3.

**Figure 11:**
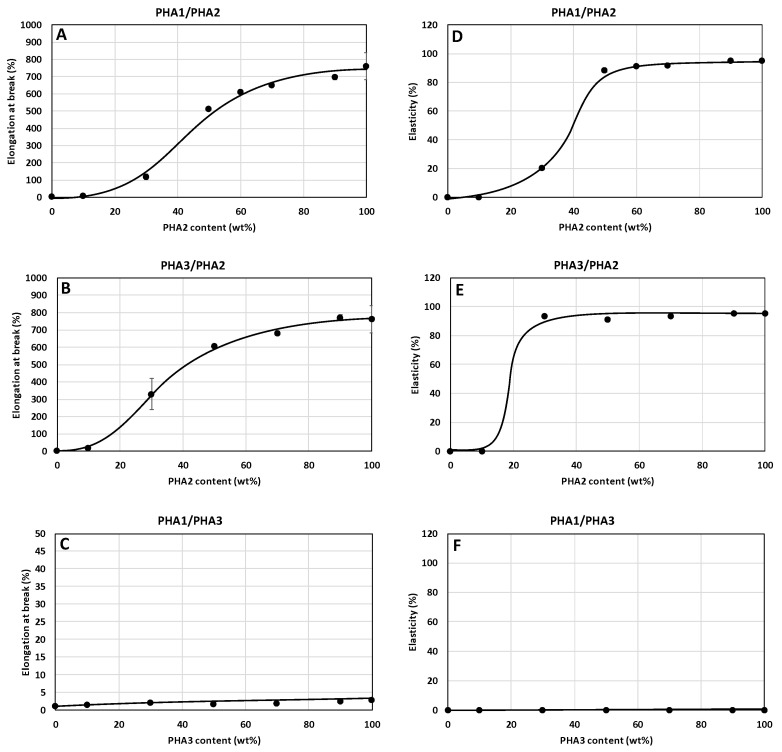
Dependence of elongation at break (**A**–**C**) and elasticity (**D**–**F**) of non-oriented specimens on the composition of binary polymer blends: (**A**,**D**) PHA1/PHA2, (**B**,**E**) PHA3/PHA2, and (**C**,**F**) PHA1/PHA3.

The dependencies of the mechanical properties of the unoriented test specimens are shown in Figure 9, Figure 10 and Figure 11. Hardness and tensile strength decrease directly proportionally with the increasing content of amorphous PHA2 in the binary blends (Figure 9A,B,D,E), while in the blend of the two semicrystalline PHAs (PHA1 and PHA3), both properties remain practically unchanged. The tensile modulus of the PHA1/PHA2 and PHA3/PHA2 blends decreases sharply with increasing content of amorphous PHA2, which is consistent with the conclusions on the miscibility and plasticizing efficiency of PHA2 in the blends, as well as with the crystallinity content. For the blend of the two semicrystalline PHAs, the tensile modulus decreases gradually with the increasing proportion of the less crystalline PHA3.

The relative elongation at break, as well as the elasticity of the unoriented test specimens, are not exhibited, or are very low, unless a two-phase system is formed in which one phase is semicrystalline and the other phase is amorphous. In accordance with the findings based on Tg values determined by DSC, relative elongation at break as well as elasticity, are observed for the binary blends PHA1/PHA2 and PHA3/PHA2 from 30% PHA2 content in the blend, which corresponds to the formation of a two-phase morphological arrangement of the blend. The binary blend PHA1/PHA3, due to the absence of a separated amorphous polymer phase, shows very low relative elongation at break and practically no elasticity.

#### 3.3.2. Mechanical Properties of Stretched Samples

**Figure 12:**
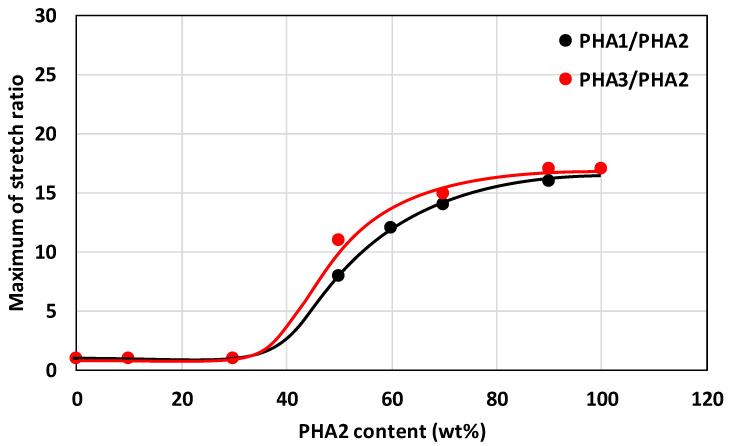
Dependence of the maximum stretch ratio on the composition of binary polymer blends PHA1/PHA2 and PHA3/PHA2. Black points and lines correspond to PHA1/PHA2, and red points and lines correspond to PHA3/PHA2.

Monofilaments of all the prepared binary blends were drawn to the maximum achievable draw ratio. The maximum draw ratio attainable for the individual binary blends, depending on their composition, is shown in Figure 12, while the PHA1/PHA3 blends could not be drawn under any conditions. Drawing of the monofilaments was possible only for those blends that exhibited a two-phase arrangement with one amorphous and one crystalline phase, with at least partial miscibility in the amorphous phase. According to the DSC-determined Tg values, this corresponded to the binary blends of semicrystalline and amorphous PHAs, i.e., PHA1/PHA2 and PHA3/PHA2 blends.

The binary blends of the two semicrystalline PHAs (PHA1/PHA3) did not show the formation of a miscible phase in the Tg dependencies; only a slight mutual plasticizing effect of the amorphous regions of the semicrystalline polymers occurred. At PHA2 contents above 30% in the blend, the draw ratio reached relatively high values, up to 17:1.

**Figure 13:**
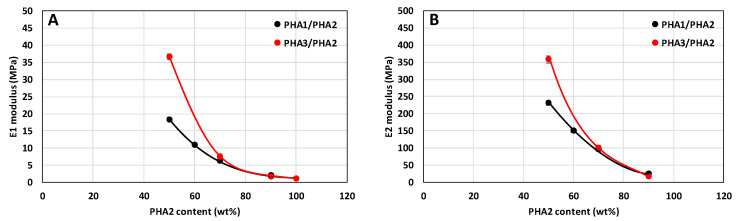
Elastic modulus E1 (**A**) and E2 (**B**), defined according to Figure 2, as a function of the composition of drawn monofilaments of binary polymer blends PHA1/PHA2 and PHA3/PHA2. Black points and lines correspond to PHA1/PHA2, and red points and lines correspond to PHA3/PHA2.

The evaluation of elastic behavior according to the procedure described in Section 2.2.7. (Determination of the Range of Elastically Reversible Deformation (RED) in Tension and E1 and E2 Moduli) was logically possible only for the drawn samples, i.e., only for the binary blends PHA1/PHA2 and PHA3/PHA2. The dependence of the moduli E1 and E2 is shown in Figure 13.

The modulus E2 could not be evaluated for the pure polymer PHA2, since the tensile curve did not exhibit the typical S-shaped form as shown in Figure 2. From Figure 13, a pronounced influence of the blend composition on both moduli E1 and E2 can be observed, where modulus E1 characterizes the resistance of the material to deformation within the region of elastic reversible deformation, while modulus E2 represents the resistance to deformation beyond the limit of maximum elastic deformation.

**Figure 14:**
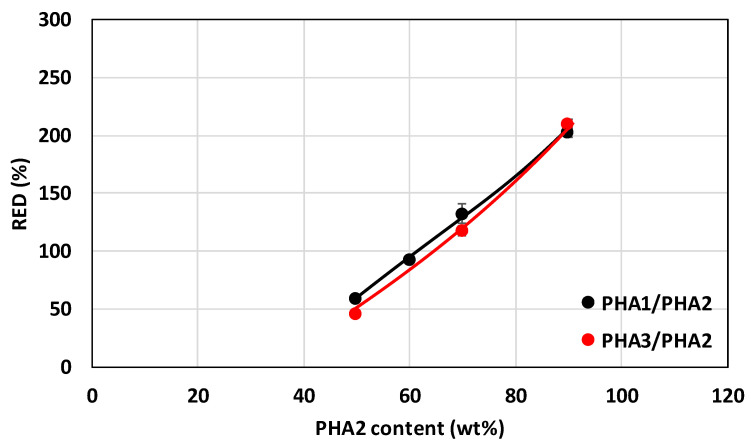
Range of elastically reversible deformation (RED) of drawn monofilaments as a function of blend composition for PHA1/PHA2 and PHA3/PHA2 systems. Black points and lines represent PHA1/PHA2 blends, while red points and lines represent PHA3/PHA2 blends.

**Figure 15:**
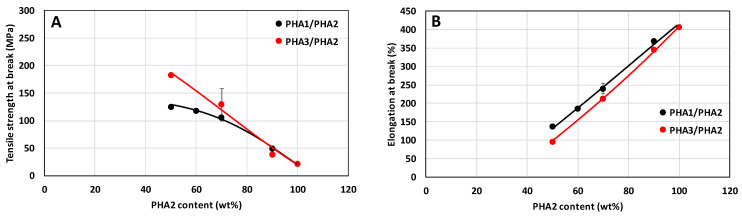
Tensile strength at break (**A**) and elongation at break (**B**) of drawn monofilaments as a function of blend composition for PHA1/PHA2 and PHA3/PHA2 systems. Black points and lines represent PHA1/PHA2 blends, while red points and lines represent PHA3/PHA2 blends.

The extent of reversible elastic deformation of the drawn monofilaments and its dependence on the composition of the binary blends are shown in Figure 14, and the mechanical properties at break are shown in Figure 15.

As expected, an increase in the range of elastic deformation corresponds to a decrease in the E1 modulus, and vice versa. The investigated blends of commercially available PHAs (PHA1, PHA2, and PHA3) exhibited thermoplastic elastomer (TPE)-like behavior at selected compositions. Elastic deformation ranges of approximately 50–200% were achieved, together with relatively high tensile strengths exceeding 150 MPa for the PHA3/PHA2 blends, and elongation at break values between 100 and 400%. Unlike earlier studies [15,20,21,23,27], these elastic properties were obtained without chemical modification of the base PHAs, the synthesis of special copolymers, or the use of crosslinking and/or dynamic vulcanization. This finding is consistent with the work of Ďurfina et al. [37], who also demonstrated highly flexible PHA blends without chemical modification. Importantly, the PHA-based blends developed in this study are fully biodegradable under natural conditions and may also be recyclable, as they are simple mechanical blends of thermoplastic components.

### 3.4. Wide-Angle X-Ray Scattering

**Figure 16:**
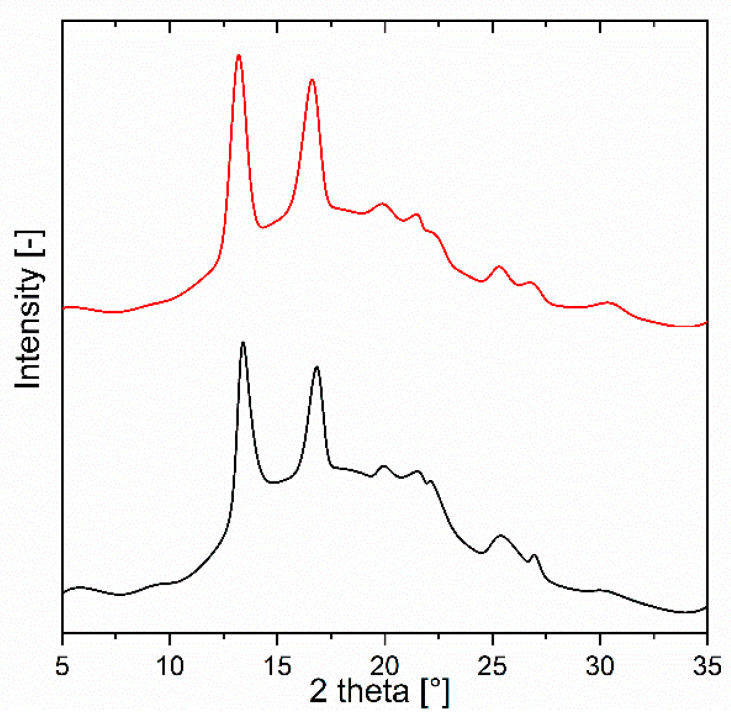
WAXS diffractograms of specimens B17 before (black) and after (red) stretching.

WAXS analysis was conducted to examine the supermolecular structure of specimen B17 before and after orientation. Mixture B17 was selected as representative due to its very promising composition in relation to mechanical properties. For polyhydroxybutyrate (PHB), the most common crystalline form is the α-phase, typically produced under melt, cold, or solution crystallization conditions. A minor fraction of the β-phase may also be induced, particularly under tensile stress applied to an oriented α-phase [60]. The α-phase forms an orthorhombic unit cell, with its two most prominent diffraction peaks appearing at 2θ = 13.5° (020) and 16.8° (110) [61]. These peaks are clearly visible in Figure 16, which presents the WAXS diffractograms of specimen B17 before and after stretching. Additional weaker and broader α-phase reflections were observed at 2θ = 21.4° (101), 22.5° (111), 25.6° (121), 27.2° (040), and 30.2° (002) [62]. A distinct peak at 2θ = 19.8° (021) was assigned to β-phase crystals [61].

As shown in Figure 16, the diffractograms of the non-oriented and oriented samples are highly similar. The calculated crystallinity values were nearly identical—25% and 26%, respectively—consistent with DSC results (Figure 6A), where crystallinity was determined as 25.47%. Both DSC and WAXS confirmed that crystallization occurs exclusively in the PHA1 (P3HB) segments of the blends. Orientation, therefore, had no significant effect on the overall degree of crystallinity and did not induce additional crystal formation (e.g., orientation crystallization). The observed improvements in elastically reversible deformation and mechanical properties can thus be attributed to morphological changes within the amorphous phase rather than alterations in crystalline structure. However, the orientation of crystals cannot be completely avoided and requires further study.

**Figure 17:**
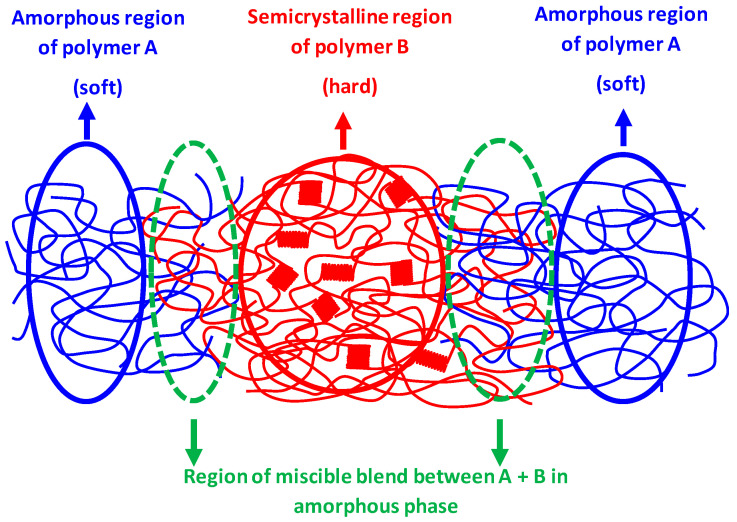
Schematic illustration of the phase structure of a binary polymer blend showing TPE behavior, where miscible segments of polymers A and B form an interphase within the amorphous phase.

Based on the presented study, it was found that for the preparation of such materials it is necessary that the blended polymers must be partially miscible in the amorphous phase and that they form a three-phase structure, where one phase is highly crystalline and the second is amorphous. The crystalline structure of the highly crystalline phase acts as the hard phase in TPEs, while the amorphous structure of the second polymer acts as the soft phase. The dynamic vulcanization or crosslinking between the two phases to ensure the elasticity of the material is substituted in our materials by the third phase, which is formed by a single phase consisting of a partially miscible blend of the amorphous regions of both polymers. A schematic representation of the phase arrangement of such a blend is shown in Figure 17.

**Figure 18:**
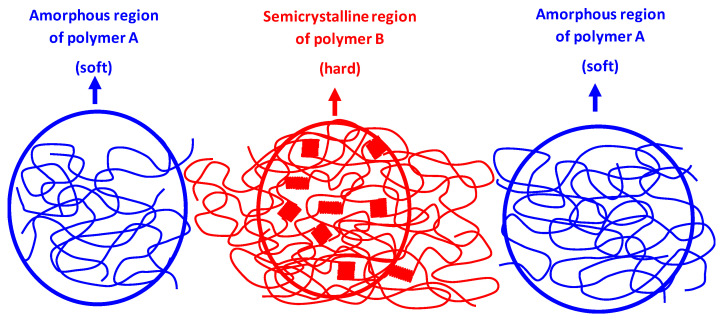
Schematic illustration of the phase structure of a binary polymer blend which do not show TPE behavior, where segments of polymers A and B are not miscible and do not form an interphase between the amorphous and semicrystalline polymers.

In our research work, PHA2 was used as the amorphous polymer A (soft phase), while PHA1 or PHA3 was used as the semicrystalline polymer B (hard phase). Partial miscibility at the interface between the hard and soft phases is a necessary condition for the elastic behavior of the material. If the components were immiscible, the hard and soft components would be separated as shown in Figure 18, and under mechanical stress, plastic flow would occur at the phase boundary, which is typical behavior of thermoplastic non-elastomeric blends and TPE behavior will not appear.

**Figure 19:**
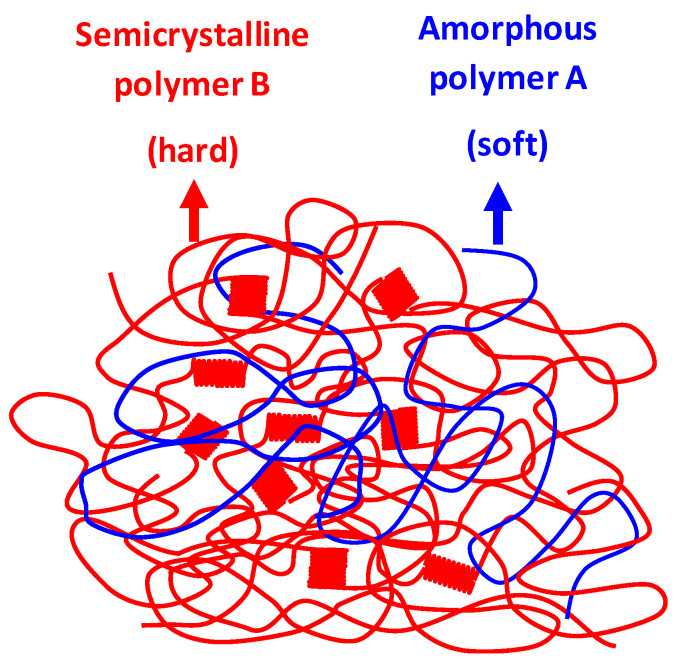
Schematic illustration of the phase structure of a binary polymer blend of polymers A and B with complete miscibility of the amorphous phase components over the entire composition range.

On the other hand, if polymers A and B were miscible over the entire concentration range, elastic behavior would again not be possible due to the absence of a highly mobile amorphous part of the blend, since it would be fixed by the crystalline structure of the semicrystalline polymer, as illustrated in Figure 19.

## 4. Conclusions

Binary blends of semicrystalline and amorphous polyhydroxyalkanoates (PHAs), prepared by melt mixing, can exhibit thermoplastic elastomer (TPE)-like properties. Elastomeric behavior originates from a two-phase morphology, where the crystalline domains of the semicrystalline PHA are coupled with the amorphous domains of the amorphous PHA through partial miscibility of their amorphous fractions. Notably, complete miscibility was not favorable; elastic properties emerged only when the amorphous PHA content exceeded ~30 wt%.

By selecting appropriate PHA types and adjusting their relative proportions, mechanical performance could be tailored. The resulting blends displayed reversible elastic deformations of 50–200%, elastic moduli of 1–40 MPa, and ultimate tensile strengths up to 200 MPa. Unlike previously reported strategies requiring copolymerization, crosslinking, or dynamic vulcanization, these properties were achieved with commercially available PHAs without chemical modification.

These findings demonstrate the potential of PHA-based blends as fully biodegradable and potentially recyclable elastomeric materials suitable for sustainable applications. In comparison with other bio-based elastomer development approaches—such as reactive blending, grafting, or crosslinking—the presented method offers several advantages: it uses commercially available, fully biodegradable PHAs, requires no chemical modification, and relies on simple melt processing compatible with conventional industrial equipment, which enhances scalability and environmental sustainability.

However, certain limitations should also be noted. Mechanical performance and elastic recovery depend strongly on blend composition and processing conditions, and the long-term thermal and mechanical stability under repeated deformation has yet to be fully assessed. Further optimization may be necessary to achieve the performance levels of chemically crosslinked elastomers.

Potential applications of these PHA-based TPE-like materials include biodegradable flexible packaging, biomedical devices, wearable components, and 3D printing filaments. Future studies should focus on scaling up processing, establishing processing–structure–property correlations, and evaluating biodegradation and aging behavior under real environmental conditions to confirm long-term performance and sustainability. Overall, this work provides a simple and sustainable pathway toward developing fully biodegradable elastomeric materials with tunable mechanical performance.

## Figures and Tables

**Table 1:** The composition of studied binary blends.

Sample	PHA1 (wt%)	PHA2 (wt%)	PHA3 (wt%)
B1	100	-	0
B2	90	-	10
B3	70	-	30
B4	50	-	50
B5	30	-	70
B6	10	-	90
B7	0	-	100
B8	-	100	0
B9	-	90	10
B10	-	70	30
B11	-	50	50
B12	-	30	70
B13	-	10	90
B7	-	0	100
B1	100	0	-
B14	90	10	-
B15	70	30	-
B16	50	50	-
B17	40	60	-
B18	30	70	-
B19	10	90	-
B8	0	100	-

**Table 2:** Surface tension of PHA polymers and interfacial tension in their binary blends.

Polymer or Polymer Pair	Surface Tension (mN/m)		Interfacial Tension mN/m
	Dispersive	Polar	
PHA1	41.33	8.79	-
PHA2	40.5	8.45	-
PHA3	39.58	7.42	-
PHA1/PHA2	-	-	0.015
PHA3/PHA2	-	-	0.077
PHA1/PHA3	-	-	2.103

## Data Availability

The original contributions presented in this study are included in the article. Further inquiries can be directed to the corresponding author.
